# How Follicular Dendritic Cells Shape the B-Cell Antigenome

**DOI:** 10.3389/fimmu.2016.00225

**Published:** 2016-06-21

**Authors:** Jan Kranich, Nike Julia Krautler

**Affiliations:** ^1^Institute for Immunology, Ludwig Maximilian University Munich, Munich, Germany; ^2^Institute of Microbiology, ETH Zurich, Zurich, Switzerland

**Keywords:** follicular dendritic cells, tertiary lymphoid organs, antigen, B-cell responses, antigenome, germinal centers, antigen trapping

## Abstract

Follicular dendritic cells (FDCs) are stromal cells residing in primary follicles and in germinal centers of secondary and tertiary lymphoid organs (SLOs and TLOs). There, they play a crucial role in B-cell activation and affinity maturation of antibodies. FDCs have the unique capacity to bind and retain native antigen in B-cell follicles for long periods of time. Therefore, FDCs shape the B-cell antigenome (the sum of all B-cell antigens) in SLOs and TLOs. In this review, we discuss recent findings that explain how this stromal cell type can arise in almost any tissue during TLO formation and, furthermore, focus on the mechanisms of antigen capture and retention involved in the generation of long-lasting antigen depots displayed on FDCs.

Follicular dendritic cells (FDCs) are cells of stromal origin that are indispensable for secondary lymphoid organ (SLO) and tertiary lymphoid organ (TLO) development and maintenance. They are located in the central region of primary follicles and in the light zone of germinal centers [GCs; (1, 2)]. Their most striking feature is the ability to capture and retain native antigen. This was first observed in 1965, when Mitchell and Abbott analyzed the location of iodine-125 labeled flagella of *Salmonella Adelaide* in draining lymph nodes of mice using high-resolution electron microscopic autoradiographs (3). Since then, the role of FDCs as crucial players in antibody responses has been widely accepted. Their main function being the presentation of native antigen, in the form of immune complexes (ICs), to B cells, thereby driving their affinity maturation during the GC reaction.

In this review, we focus first on recent findings that help to explain, how FDCs can arise in almost any tissue undergoing TLO formation and, second, on their ability to retain antigen in B-cell follicles. For a more detailed description of FDC biology, we refer the reader to other recent reviews (4, 5).

## Requirements for FDC Development

After the first mentioning of FDCs little more than half a decade ago, initial experiments, mainly using bone marrow chimeras (6, 7), indicated that FDCs are of stromal, radioresistant, and likely sessile character. In the meantime, extensive data were brought forward attributing important functions to FDCs in B-cell responses, such as the provision of the chemokine CXCL13, essential to allure B cells into the follicles in a CXCR5-dependent manner (8). Interestingly, the dependence of B cells and FDCs was found to be mutual; in the absence of B cells, FDCs did not form (9). B cells were shown to be the main source for lymphotoxins (LT) and tumor necrosis factors (TNF), which upon binding to their respective receptors, LTβR and TNFR1, present on the surface of FDCs and their precursors, acted as potent drivers of FDC maturation (9–16). Furthermore, after the initial generation of FDCs sustained LT signaling was shown to be required for keeping them in a differentiated and functional state (17).

While it was soon recognized that FDCs are a central component of B-cell follicles in spleen and in lymph nodes, their appearance was not limited to SLOs. FDCs were also shown to contribute to non-encapsulated lymphoid structures, such as the isolated lymphoid follicles of the intestine (18). In addition to this, FDCs were frequently observed during certain chronic inflammations in non-lymphoid tissues. As a result of an unresolved inflammation during autoimmunity (e.g., rheumatoid arthritis) or during chronic infections (e.g., hepatitis C infection), such tissues can undergo remodeling into TLOs (19–21), containing FDCs and microanatomically segregated T and B cell areas. Autoimmune diseases and chronic inflammations with FDC involvement are summarized in Table 1. The notion that FDCs can possibly be generated everywhere in the body suggests that their precursors sport either considerable motility or that they are derived from a non-migratory ancestor. Bone marrow chimera experiments, where FDCs in spleen and LN were generated from host cells, added evidence to the latter hypothesis (6, 7). The idea that FDCs could have differentiated from a local precursor, was further supported by the finding that FDCs shared markers with other stromal cells of SLOs and TLOs and showed similarities with fibroblasts and mesenchymal cells (1, 22, 23). In parabiont experiments, where the blood circulation of two mice was surgically connected for 3 months, no FDCs had been generated from the surgically attached counterpart (24). This also corroborated a model of a non-migratory and rather local precursor, giving rise to FDCs.

**Table 1:** **Human diseases with lymphoid neogenesis**.

**Autoimmune diseases**	**Chronic allograft rejection**
Rheumatoid arthritis (88–91)	Organ transplantation (118, 119)
Hashimoto’s thyroiditis and Graves’ disease (92–95)	
Myasthenia gravis (96–98)	**Other chronic inflammations**
Sjogren’s syndrome (99–101)	Ulcerative colitis (120, 121)
Multiple sclerosis (102–104)	Atherosclerosis (122, 123)
Cryptogenic fibrosing alveolitis (105, 106)	
Systemic lupus erythematosus (107, 108)	**Cancer**
	Non-small cell lung cancer (124, 125)
**Infectious diseases**	Colorectal carcinoma (126)
Chronic hepatitis C (109, 110)	Ductal breast carcinoma (127, 128)
*Helicobacter pylori*-induced gastritis (111–115)	Melanoma (metastasis) (129)
Chronic Lyme disease (116, 117)	Mucosal-associated lymphoid tissue lymphoma (115)

In a murine model of chronic inflammation, transgenic overexpression of LTα under the rat insulin promoter (RIP-*Lta*) leads to the formation of TLOs in kidneys, including fully matured FDCs (25–27). When these mice were treated with LTβR-Ig decoy receptors (17) to remove mature (renal) FDCs followed by transplantation of their kidneys into recipient mice, this led to the reformation of FDCs exclusively derived from cells of the transplanted donor kidneys. This finding proved that, even during the generation of TLOs, FDC precursors are tissue-intrinsic (25). Detailed analysis of the expression of the FDC-expressed molecule Mfge8 [FDC-M1; (28)] during splenic organogenesis as well as in mice lacking FDCs, further suggested that the earliest FDC precursor was located in the splenic perivascular space. These putative precursors expressed PDGFRβ and SMA. Since mature FDCs do not express PDGFRβ, lineage-tracing experiments (using Pdgfrb-Cre mice) were performed and confirmed that FDCs had derived from such PDGFRβ-positive precursors. The expression of PDGFRβ and SMA as well as their localization indicated that these cells were in fact mural vascular cells. Depending on the localization (surrounding small capillaries or larger vessel) and their appearance, mural cells are divided into single-layered pericytes or several layers of vascular smooth muscle cells. Mural cells can be isolated from the stromal-vascular fraction of white adipose tissue (29). The transplantation of PDGFRβ-positive cells, sort-purified from the stromal-vascular fraction, into the kidney capsule of mice lacking endogenous FDCs gave rise to artificial lymph nodes containing fully differentiated FDCs. This showed that FDCs are generated from perivascular cells. The ubiquity of such perivascular cells and, therefore, likely FDC precursors also explains why it is possible for FDCs to arise in any tissue or organ (25). It remains to be shown, whether any mural cell can give rise to FDCs or whether it needs to be derived from specific tissues, such as the adipose tissue. This is of particular interest as LN anlagen usually are inserted within fat pads. Indeed stimulation of LTβR signaling inhibits adipocyte differentiation and promotes a fibroblast-like phenotype (30).

**Figure 1:**
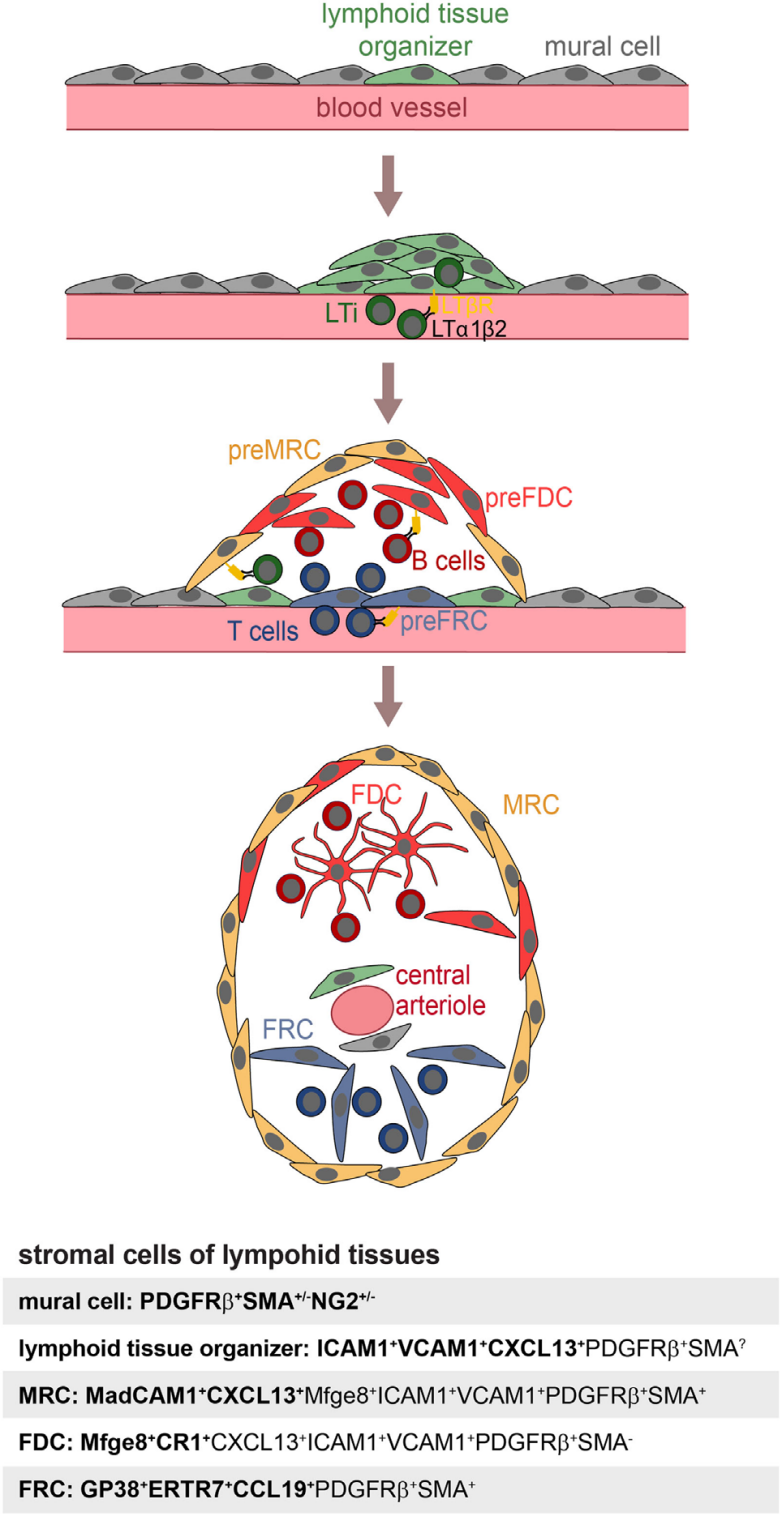
**Model of FDC development**. Mural cells and lymphoid tissue organizer cells line blood vessels (top panel) at places where future lymphoid tissues develop. The arriving lymphoid tissue inducer cells (LTi) express membrane bound LTα1β2 and trigger expansion of lymphoid tissue organizer cells and upregulation of the chemokine CXCL13 (second panel). Further recruitment of T and B lymphocytes provisioning LTα1β2 leads to the induction of marginal reticular cells (MRC), follicular dendritic cell (FDC), and fibroblast reticular cell (FRC) precursors, all likely to be generated from lymphoid tissue organizer cells (third panel). Shared expression of markers of MRC and FDC suggest a close lineage relationship, further supported by appearance of mature FDC next to MRC. The influx of T and B cells further leads to a zonal segregation and differentiation of blood vessel into marginal sinus and central arteriole as shown in case of the spleen (bottom panel). Markers used to identify specific stromal populations are highlighted in bold.

Follicular dendritic cells are not the sole stromal cell of SLOs. Fibroblast reticular cells (FRCs) contribute to the structure and function of the T-cell zone, while marginal reticular cells (MRCs) are important for the function and the structure of the splenic marginal zone (MZ) (31). Recently, novel stromal subpopulations were identified, such as the versatile stromal cells at the T cell–B cell border of inflamed B-cell follicles [VSCs; (32)] and the CXCL12-expressing reticular cells of the GC dark zone [CRCs; (2)]. FDCs, MRCs, and FRCs share the expression of many markers, such as LTβR, BP-3, VCAM-1, and ICAM-1 (25, 33–35), which could also suggest a common precursor. To identify this potential precursor, labeling experiments were performed with fetal mesenchymal progenitors of spleen and lymph nodes. Splenic mesenchymal precursors were followed using either Nkx2-5-Cre or Islet1-Cre reporter mice and found to contribute to FDCs, FRCs, MRCs, and mural cells (36). A reporter mouse for neural crest cells (Wnt-1-Cre), embryonic progenitor cells that give rise to mesenchymal structures of the head and the neck region, was used to test if FDCs in auricular and cervical lymph nodes were derived from such cells. Indeed, Jarjour et al. could show that FDCs as well as MRCs and other stromal cells can be labeled with this technique (24). While the authors did not confirm if the Wnt-1-Cre reporter also labeled PDGRβ^+^SMA^+^ perivascular precursors in lymph nodes, FRCs, and precursors thereof, have been attributed a pericyte-like character and reside as CCL21^+^CCL19^+^PDGFRβ^+^SMA^+^ cells in perivascular locations of inguinal and popliteal lymph nodes (35, 37). The transplantation of fetal splenic Nkx2-5-reporter positive cells or adult adipose PDGFRβ stromal vascular cells generated artificial lymph nodes, further supporting the idea that these early precursors can contribute to all stromal compartments and even includes stromal organizer cells able to initiate lymph node anlagen (25, 36). A model for FDC development is illustrated in Figure 1.

## The Discovery of FDCs

As mentioned above, the deposition of antigen within SLOs was studied extensively in the 1960s, using radioactively labeled microbial antigens, such as isolated flagellin derived from *Salmonella*. Immunofluorescent detection of antigens, which was a very new technique at that time, was also used in some of the studies (38, 39). A common observation was that even though most of the antigen was endocytosed by phagocytic cells, some remained extracellularly on the surface of cells, whose identity was obscure at that time. Miller and Nossal described that within the follicle cell surface-bound antigen was trapped on fine processes of cells, which at that point they believed to be a phagocytic cell subset (39). However, later electron-microscopy studies clarified that antigen was rather associated with the dendritic processes of non-phagocytic reticular cells and that these cells formed large web-like structures (3, 40, 41).

While further studies in the following years dealt with the exact distribution of antigen within the lymph node and GCs, the precise nature of these antigen-retaining reticular cells remained unclear for several more years. Various different names were used for these cells by the different laboratories that studied them. So they were also sometimes referred to as dendritic macrophages or dendritic reticular cells (40, 42). However, the common feature recognized by all these studies (43) was the extraordinary ability of these cells to retain antigen on their cell surface. Hence, these cells clearly differed from the typical phagocytic cells. In 1978, Chen et al. published a detailed anatomical and functional study of these cells. They introduced the name “FDCs” owing to their long cytoplasmic processes, and not because of relations to classical dendritic cells (DCs) (44). The authors realized that the name may not be ideal and suggested that at a later time point, when more would become known about these cells the name might need to be reconsidered (44). However, even when it became evident that FDCs lacked MHC class II expression, a molecule expressed at high levels by conventional, hematopoietic DCs, the name FDCs persisted (45) with the consequence that FDCs are still often confused with conventional DCs.

Using electron-dense tracers, Chen et al. showed that FDCs, unlike macrophages, do not actively endocytose (43, 44), a view that has recently been challenged by a study that showed that FDCs endocytose ICs, which they acquire from non-cognate B cells. In contrast to macrophages, ICs endocytosed by FDCs retain their native form and recycle to the cell surface (46), a feature essential for long-term antigen display. Electron microscopy further revealed that FDCs have unique cellular structures, including large, irregular nuclei, containing little heterochromatin, and only few organelles. One striking feature was that FDCs only had small cell bodies, while their cytoplasm extended into many filiform dendrites, forming an extensive net-like structure, which seemed to act like a cap covering the secondary follicle (43, 44).

## Immune-Complex Trapping – The Cardinal Function of FDCs

In the 1960s, researchers tried to address the molecular requirements for antigen retention in B-cell follicles. Nossal et al. compared antigen distribution in non-immunized rats with those that either had received a passive or an active immunization against *Salmonella* prior to administration of radiolabeled *Salmonella* flagellin. Strikingly, they observed that immunization greatly influenced the distribution of antigen within the lymph node. Rats that were actively or passively immunized before they received radiolabeled antigen had a faster and more intense accumulation of antigen in their follicles than non-immunized animals. The increase in follicular antigen deposition seen in immunized rats led the authors to conclude that an opsonin was responsible for the efficient targeting of antigen to the follicle, and that this opsonin was likely to be an antibody (47). This observation was also confirmed to hold true in other species: Humphrey et al. immunized rabbits with non-microbial antigens (radiolabeled hemocyanin or human serum albumin). Prior to injection of radiolabeled antigen, the rabbits were either immunized with a single injection of unlabeled antigen, received repeated injections of antigen shortly after birth (inducing antigenic tolerance) or had remained untreated (naive). While uptake of radiolabeled antigen by medullary sinus macrophages did not differ between the three treatments, no antigen was retained by FDCs in the follicles of naive rabbits. Furthermore, tolerized rabbits had no detectable levels of antibody and showed no follicular antigen retention by FDCs. Thus, it was established that for the follicular retention of antigen the presence of antigen-specific antibodies was crucial (48).

Still, some studies had shown that low-level retention of antigen also occurred in non-primed animals (47). Hence, some doubts remained, whether the “follicular opsonin” was the antibody itself or if another, antibody-induced substance, was involved.

Experiments by Williams then showed that a substance produced by lymphocytes was important: he had previously seen a diminished uptake of *Salmonella* flagellin in lymphoid follicles after depletion of peripheral lymphocytes by partial irradiation with shielded bone marrow (49). This observation had led him to assume that lymphocytes produced substances with opsonizing activity. To test this hypothesis, he monitored the accumulation of flagellin in follicles in the absence of peripheral lymphocytes and assessed how the application of normal rat serum or antibody influenced follicular antigen deposition. A decline in the retention of radiolabeled antigen was observed from day 5 after irradiation onward. Jaroslow and Nossal had previously shown that FDCs are highly resistant to irradiation, so an impairment of FDC function could be excluded as the reason for reduced antigen accumulation following irradiation (50). To restore the antigen retention, normal rat serum or anti-flagellar immune serum was injected. Immune serum significantly improved antigen trapping, as did normal rat serum, but for the latter 25-times more volume was required. By contrast, fetal calf serum did not improve the antigen uptake in follicles, showing that serum-dependent antigen trapping was species specific. Furthermore, neither injection of lymphocytes nor supernatant from cultured lymphocytes showed an effect. While this study had pitfalls mainly due to the irradiation, still an important conclusion could be drawn from this study; immune serum contained large-amounts of the “follicular opsonin,” also supporting the idea, that antibodies might be the crucial opsonin. However, the finding that non-immune serum also was able to restore antigen retention in the follicle even though at a much lower efficiency, suggested the presence of additional opsonins (49).

While it became generally accepted that antigen–antibody complexes were crucial for efficient targeting of native antigen to FDCs, years had to pass until other factors essential for IC-trapping, namely complement, were identified. Only in 1974 Pepys found that depletion of the complement component C3 by cobra venom factor, strongly reduced T-cell-dependent B-cell responses to sheep red blood cells (SRBC), illustrating the central role of C3 in the induction of antibody production (51). One year later evidence that complement was required to retain antigen in the GC came from Papamichail et al., who reported that complement inhibition with cobra venom factor blocked trapping of aggregated IgG in the splenic follicle (52). In line with this, Klaus and Humphrey observed that chronic depletion of C3 inhibited memory B-cell formation and concluded that the assembly of an antigen–antibody–C3 complex on FDCs is crucial for B-cell memory (53). More than 20 years later, the complement receptors 1 and 2 (CR1, CR2) were found to be responsible for capturing of C3-containing ICs (54). In humans, two separate genes encode for CR1 and CR2; in mice, however, the Cr2 locus encodes for both CR1 (CD35) and CR2 (CD21) and expression of either CR1 or CR2 is determined by alternative splicing. CR2 binds degradation products of C3, such as iC3bm C3d,g, C3d, while CR1 binds C3b and C4b (55). All mature B cells express CR2, but particularly high levels are found on MZ B cells. FDCs predominantly express CR1 (56). On B cells, CR2 acts as a B-cell receptor (BCR) co-receptor. Fusing antigen with one or more copies of C3d lowered the amount of antigen needed to induce B-cell responses up to 10,000 fold in a CR2-dependent manner (57). Several studies have shown that FDCs utilize CR1/2 to retain antigen on their surface (54, 56, 58, 59).

In addition to complement receptors, FDCs use other receptors to bind ICs. *Ex vivo* IC-trapping experiments on splenic cryosections of immunized mice were tested in presence or absence of serum for the retention of ICs. In presence of serum, most trapping depended on CR1/2, since CR1/2 blocking antibodies dramatically reduced IC-capturing. However, in absence of complement (without serum), some trapping on a subset of FDCs still occurred. This residual trapping could be blocked with anti-FcγRIIβ antibodies (59). The importance of Fc-receptors for IC-trapping by FDCs was also confirmed *in vivo*, since *Fcgr2b^−^*^/^*^−^* mice showed significantly reduced IC-trapping, and although primary antibody responses are unaltered in mice with FcγRIIβ-deficient FDCs, recall responses are diminished (60).

In summary, the crucial components to deliver antigen to FDCs are antigen-specific antibodies and complement factors. But how exactly antigen reaches FDCs has remained unclear for a long time.

## Mechanisms of Antigen Delivery to FDCs

Already in 1983, Szakal et al. described antigen transport cells (ATCs) that supposedly transported antigen from the subcapsular sinus to the FDCs. These cells were non-phagocytic and had morphological similarities with FDCs, leading to the assumption that these cells might be pre-FDCs, an observation which remained unconfirmed (61, 62).

**Figure 2:**
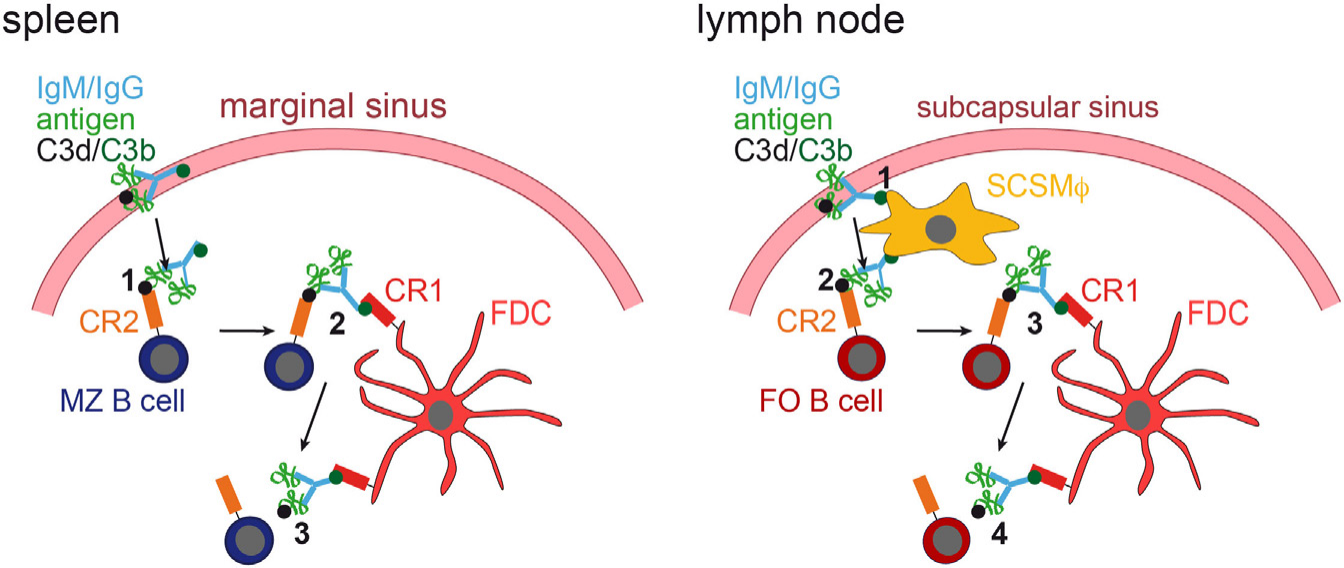
**IC acquisition**. In the spleen (left panel), non-cognate marginal zone (MZ) B cells capture ICs, consisting of antigen, IgM or IgG, and C3 degradation products (C3d and C3b), from the blood stream (1). MZ B cells, which have captured ICs in a C3d/CR2-dependent fashion, then migrate into the follicle, where they transfer the ICs onto FDCs, which bind them via C3b and CR1 (2). The ICs are then released from the MZ B cell (3). In the lymph node (right panel), subcapsulary sinus macrophages (SCSMϕ) capture ICs consisting of antigen, IgM or IgG, and C3d and C3b degradation products from the lymph (1). SCSMϕ migrate into the follicle and transfer ICs to follicular (FO) B cells in a CR2-dependent manner (2). Subsequently, FO B cells transfer the ICs onto FDCs (3, 4).

It was shown that in the spleen MZ B cells capture IgM-containing immune complexes (IgM-ICs) and transport and deposit them onto FDCs within the B-cell follicle (63, 64). This transfer was dependent on complement and CR1/CR2, and mice deficient for those factors, showed no accumulation of IgM-ICs on FDCs. Still, how would antigen be brought to FDCs in lymph nodes that lack MZ B cells? This was revealed by two-photon microscopy studies (65, 66). Phan et al. showed that in lymph nodes subcapsulary sinus (SCS) macrophages capture immunofluorescently labeled ICs [Phycoerythrin:ICs; (65)]. These macrophages monitor the lymph fluid that arrives in the subcapsular sinus, bind large amount of ICs and have little endocytic activity. ICs travel along the processes of these macrophages and transfer antigen onto non-cognate follicular B cells in a complement receptor-dependent manner. Subsequently, ICs are shuttled from the B cells onto FDCs (66). Mechanisms of IC delivery to FDCs are depicted in Figure 2.

## The Role of FDC-Bound ICs in B-Cell Responses

Immune complexes bound by FDCs are organized in a bead-like formation, as the so-called iccosomes. These IC-coated bodies can be endocytosed by tingible body macrophages (TBMϕs) and B cells (67). The effect on B-cell activation, GC development, affinity maturation, and memory B-cell maintenance of FDC-bound ICs has been studied in great detail (1). It is generally accepted that FDC function as storage of native antigen. During the GC reaction, high-affinity B cells access antigen, internalize, process, and display it to T helper cells, thereby receiving BCR stimulation as well as additional T helper cell-derived survival signals (68, 69). Early studies assessing the influence of FDCs on B-cell activation were performed *in vitro* using FDC-enriched clusters. They showed that only in the presence of FDCs, ICs (in the form of isolated iccosomes) were able to strongly activate B cells, evidenced by substantially increased antibody production against the cognate antigen. Hence, FDCs stimulate B cells via FDC-bound antigen, but also via antigen-independent FDC products (70). In addition to this, Boes et al. found that in the absence of secreted IgM antigen trapping by FDCs was reduced and GC formation as well as antibody affinity maturation impaired (71).

Based on these and other studies, the view that FDCs can take part in B-cell activation and play an important role during affinity maturation by displaying native antigen and by presenting survival signals to B cells and that they are involved in memory B-cell development has become generally accepted.

However, this view has been challenged by results obtained from mice that produce only membrane bound IgM, hence, unable to make ICs. These mice showed normal GC formation, despite absent IC-trapping by FDCs (72). Furthermore, CR1/2-deficient mice are also able to form GCs and B cells of such mice even undergo affinity maturation, although numbers and size of the GCs were reduced and antibody levels much lower than those in their wild-type counterparts (73, 74). The role of FDCs and ICs trapped by them has then been critically discussed (75, 76). Haberman and Shlomchik concluded that the role of FDCs in providing non-specific support for the GC reaction is undisputed, but ICs on FDCs might only be important under certain conditions. By contrast, Kosco-Vilbois stresses that immune responses are still most efficient in the presence of ICs on FDCs. Thus, an efficient vaccine should maximize the deposition of ICs on FDCs.

## Speculations on Additional Functions of FDCs

**Figure 3:**
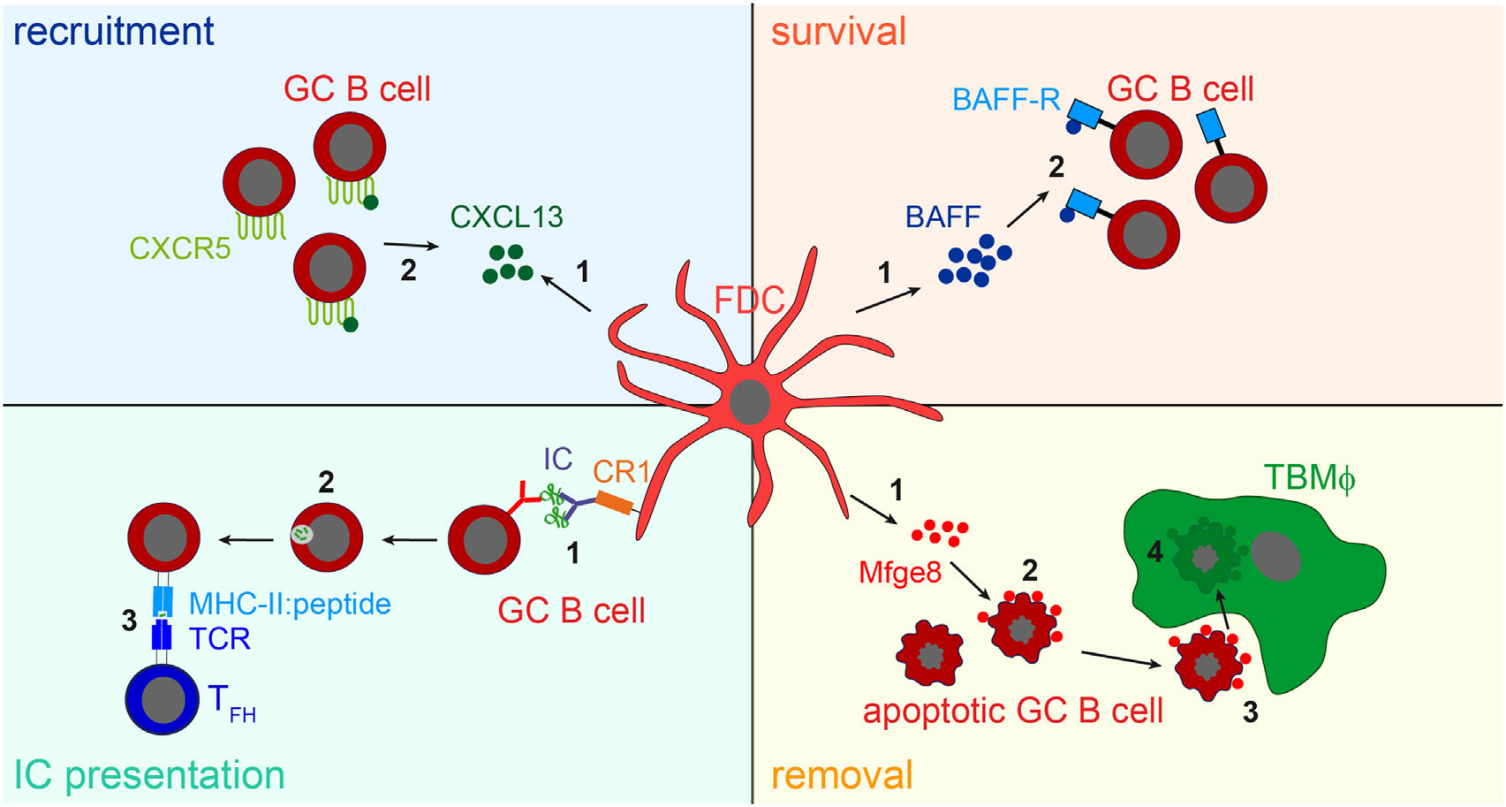
**FDC influence on B cells**. *Recruitment*: FDCs secret the B cell attracting chemokine CXCL13 (1). GC B cells express the CXCL13-binding chemokine receptor CXCR5 and are thereby attracted towards the B cell follicle (2). *Survival*: FDCs produce B-cell activating factor [BAFF, (1)], which is involved in regulating GC B cell survival (2). *IC presentation*: Via their CR1s FDCs present naive antigen to GC B cells (1). Antigen-specific GC B cells, recognizing the antigen via their BCR, endocytose, and process it into peptides (2), and subsequently present it to T follicular helper cells (T_FH_ cells) in form of peptide-MHCII (3). T_FH_ cells then supply cognate B cells with survival signals. It is assumed that after each round of somatic hypermutation, B cells with high-affinity BCRs are able to access antigen presented by FDCs and, thus are able to interact with T_FH_ cells. This leads to the positive selection of such B cells, while others bearing lower affinity receptors are unable to compete for binding to limiting amounts of antigen and undergo apoptosis. *Removal*: the large number of GC B cells that fail to bind antigen presented by FDCs and do not receive T_FH_ help die by apoptosis. To prevent autoimmunity, these cells have to be cleared efficiently. FDCs secrete the apoptotic cell binding protein Mfge8 (1). Mfge8-opsonized apoptotic cells (2) are then recognized and removed by tingible body macrophages (TBMϕs, 3, 4).

While the consequences of IC-trapping by FDCs are still not fully understood, other functions of FDCs have been identified. The expression of cytokines directs B cells to primary and secondary follicles (1, 77), they supply B cells with trophic factors, such as B-cell activating factor [BAFF; (78)] or instruct TBMϕs to remove apoptotic GC B cells through the secretion of the phosphatidylserine-binding bridging molecule Mfge8 (28). The main functions of FDCs are shown in Figure 3.

However, we still think that apart from the role of FDCs in establishing the correct follicular microarchitecture and enabling the formation of GCs (79), one of the most important functions of FDCs lies in the trapping of antigen and activation of B cells. FDCs are the only known cell type that extensively trap ICs for long periods of time in a way that protects native antigen from degradation (46). Therefore, FDCs shape the antigenome – the sum of all native antigens that can be detected by B cells, in primary and secondary follicles. However, although evidence is lacking, we postulate that FDCs might not only trap antigen in the form of ICs, but also in a way that does not require the presence of antigen-specific antibodies, hence, would allow antigen trapping also in individuals that have not previously been exposed to the antigen.

The reasons why we think a trapping mechanism independent of antibodies might exist are the following. It is still widely unknown how naive B cells are activated in a non-immune host, where ICs are absent. While in immune hosts not only the capturing of antigen by FDCs is dependent on ICs but also the antigen transport into the follicle requires ICs. It has been shown that native antigen is captured by subcapsulary sinus macrophages (SCSMϕ) in lymph nodes (66) and by MZ B cells in the spleen (63) in an IC-dependent manner. These cells then transport the antigen inside the follicle and deposit it onto FDCs, where it then can activate naive B cells.

While natural IgM is certainly of importance to control infections before high-affinity antibodies are generated (80), natural IgM does not seem to be sufficient to deposit easily detectable amounts of antigen onto FDCs (47–49). Furthermore, it is known that some viral glycoproteins (such as HIV gp120) quite successfully evade recognition by antibodies, e.g., by shielding their epitopes with glycans (81). This makes it very hard for the infected host to develop antibodies against such antigens. In such a case, it seems counterintuitive that antibody-containing ICs are required to mount a B-cell response, especially since such an IC would potentially mask the rare epitope needed for the initial BCR engagement in order to activate the cognate B cell.

Another study showed that soluble antigen readily diffuses through the follicle and is capable of activating B cells (82). However, this study used adoptively transferred BCR transgenic B cells, with the consequence that an unphysiological high number of antigen-specific B cells are located in the follicles. If soluble antigen is efficient enough to trigger B-cell responses, in a more natural setting, where antigen, as well as antigen-specific B cells are limiting, remains an open question.

Using BCR transgenic B cells specific for HEL and DCs that were pulsed with HEL, Qi et al. showed in a two-photon microscopy approach that DCs can carry unprocessed antigen into the lymph node and activate cognate B cells in extra-follicular regions (83). If this is a general mechanism of antigen transport into the lymph node and how efficiently this activates B cells under more physiological conditions remains to be addressed.

While all these possibilities are certainly able to trigger B-cell responses, the presentation of antigen via FDCs (46) seems to be the most intuitive and effective way to bring antigen in contact with antigen-specific B cells.

Secondary lymphoid organs are considered to be specialized structures to ensure that a DC, presenting pathogen-derived peptides via MHC class II molecules, finds and activates the rare cognate T cell that recognizes these peptides. Accordingly, we think that the network-like structure of FDCs within B-cell follicles ensures that a rare cognate B cell meets its specific antigen. To do so FDCs retain native antigen sufficiently long, protected from degradation and at the same time concentrating it at the location where many B cells reside. This strongly increases the likelihood of antigen-encounter by the rare cognate B cell. Such a mechanism is especially important when antigen and cognate B cells are limited. In artificial systems where large quantities of antigen are combined with a high frequency of antigen-specific B cells (like in models that use BCR transgenic B cells), naive B cells might readily get in contact with their antigen even without the need of FDCs. Thus, although, it is often assumed that FDCs play no or only a minor role in the initial priming of naive B cells (84), we, therefore, postulate that efficient mechanisms exist, which allow FDCs to capture, retain, and present antigen in non-immune hosts in an antibody-independent manner and, thus, can play an important role in the initial activation of B cells.

## Concluding Remarks

The biology of FDCs has been extensively studied, nevertheless, many questions regarding these cells remain unanswered.

Although there have been some controversies about their importance in the past (75, 76), FDCs are now generally accepted as indispensable for efficient, high-affinity antibody responses. Importantly, FDCs are the only known cell type that functions as a long-term antigen depot. We think it is important to understand what the consequences of such antigen storage are for the activation of B cells, especially during chronic inflammations, where FDC-containing TLOs arise in non-lymphoid tissues, e.g., during rheumatoid arthritis (85). There, FDCs might function as a tissue-specific depot of antigen. Although, little is known, how antigen is acquired by FDCs in TLOs, it might differ from antigen acquisition in lymph nodes or spleen. Also the nature of the antigen might be different from antigen that circulates in the blood stream and is then captured by FDCs in the spleen or from antigen that is transported by the lymph flow to the draining lymph node. It is possible that FDCs in TLOs might preferentially capture antigens that are released in the affected tissue by local tissue damage and that this drives GC formation and chronic inflammation or autoimmunity in affected tissues. Hence, FDCs have long been considered an attractive target for therapeutic intervention, e.g., by administering LTβR-Ig fusion proteins, which lead to FDC ablation (86). However, clinical trials assessing efficacy of LTβR-Ig fusion proteins (Baminercept) in RA patients did not show a measurable effect in treated patients (87). Other studies, assessing, for example, the efficacy of Baminercept to treat Sjögren’s syndrome are still ongoing (study ID NCT01552681).

Being “dynamic antigen libraries” (5), FDCs hold valuable information about antigens and antigen epitopes that trigger antibody responses. This information would be of relevance in autoimmunity, chronic inflammation, and cancers with intratumoral TLOs to identify antigenic triggers of disease or cancer antigens that can be used to fight tumors. However, there are currently no techniques available to screen and define the antigenome of FDCs. It would be important to develop techniques that allow the isolation of the FDC antigenome. Subsequent proteomic analysis of the FDC-trapped antigens would provide valuable information that could be exploited for development of novel vaccines or for therapeutics against chronic inflammation.

## Author Contributions

JK wrote the abstract, introduction, and following chapters: The discovery of FDCs, Immune complex trapping – the cardinal function of FDCs, Mechanisms of antigen delivery to FDCs, The role of FDC-bound ICs in B-cell responses, Speculations on additional functions of FDCs, and Concluding remarks. He also prepared Figures 1, 2, and 3. NK wrote the chapter “Requirements for FDC development,” and prepared Table 1 and Figures 1, 2, and 3.

## Conflict of Interest Statement

The authors declare that the research was conducted in the absence of any commercial or financial relationships that could be construed as a potential conflict of interest. The handling Editor declared a shared affiliation, though no other collaboration, with one of the authors [J.K.] and states that the process nevertheless met the standards of a fair and objective review.
